# A novel, fast, HMM-with-Duration implementation – for application with a new, pattern recognition informed, nanopore detector

**DOI:** 10.1186/1471-2105-8-S7-S19

**Published:** 2007-11-01

**Authors:** Stephen Winters-Hilt, Carl Baribault

**Affiliations:** 1 Dept. of Computer Science, University of New Orleans, New Orleans, LA 70148, USA; 2 Research Institute for Children, Children's Hospital, New Orleans, LA 70118, USA

## Abstract

**Background:**

Hidden Markov Models (HMMs) provide an excellent means for structure identification and feature extraction on stochastic sequential data. An HMM-with-Duration (HMMwD) is an HMM that can also exactly model the hidden-label length (recurrence) distributions – while the regular HMM will impose a best-fit geometric distribution in its modeling/representation.

**Results:**

A Novel, Fast, HMM-with-Duration (HMMwD) Implementation is presented, and experimental results are shown that demonstrate its performance on two-state synthetic data designed to model Nanopore Detector Data. The HMMwD experimental results are compared to (i) the ideal model and to (ii) the conventional HMM. Its accuracy is clearly an improvement over the standard HMM, and matches that of the ideal solution in many cases where the standard HMM does not. Computationally, the new HMMwD has all the speed advantages of the conventional (simpler) HMM implementation. In preliminary work shown here, HMM feature extraction is then used to establish the first pattern recognition-informed (PRI) sampling control of a Nanopore Detector Device (on a "live" data-stream).

**Conclusion:**

The improved accuracy of the new HMMwD implementation, at the same order of computational cost as the standard HMM, is an important augmentation for applications in gene structure identification and channel current analysis, especially PRI sampling control, for example, where speed is essential. The PRI experiment was designed to inherit the high accuracy of the well characterized and distinctive blockades of the DNA hairpin molecules used as controls (or blockade "test-probes"). For this test set, the accuracy inherited is 99.9%.

## Introduction

**Figure 1:**
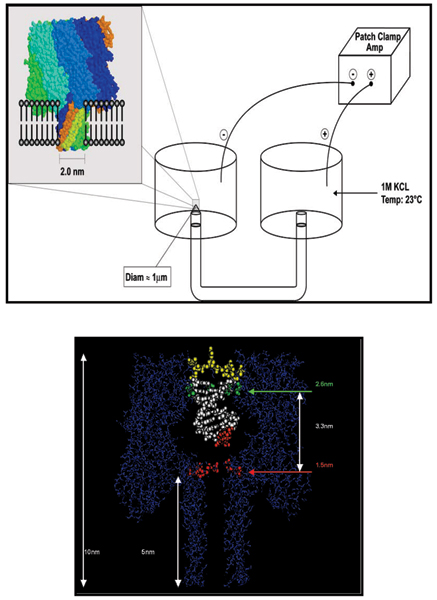
Left Panel: A lipid bilayer supports the alpha-hemolysin heptamer that creates a pore, or channel used to collect the data, as shown left. The channel is supported by an aperture, which allows the flow of ions between cis (here, left) and trans (here, right) wells. Right Panel: The assembled α-hemolysin pore shown to scale, with a captured dsDNA molecule. As shown, the double stranded form is too wide to pass through the pore, while a single strand may pass through.

It appears possible to obtain kinetic features directly from the channel blockade signals obtained during the capture of certain molecules in a nanopore detector, shown in Fig. 1 (see further details on the Detector in the Background), where individual blockade levels appear to correlate with binding or conformational states of the molecule [1-3]. The extraction of kinetic features from nanopore detector measurements, e.g., obtaining the median dwell times of the most frequented channel blockade levels, requires that we faithfully preserve the dwell times of the various blockade states (or "levels") encountered during the channel-capture event, to the exclusion of short noise pulses that might normally be misinterpreted as short dwell times. During analysis using conventional Hidden Markov Models (HMMs), both the combination of first-order modeling and pulsed noise conspire to produce premature state transitions and hence incorrect assessment of kinetic features. From the preliminary work in [4], in particular, we know that bi-level synthetic data with Poisson-distributed dwell times provides an example of such a pulsed noise instability. Here we clarify how to solve the problem with (1) dwell-time dependence in the state-to-state transitions; and (2) emission variance amplification (EVA projection); and show new experimental results.

The conventional HMM is first order and fixed (scalar) in the transition probabilities for remaining in a given state, which leads to a geometric length distribution for remaining in that state [5] – i.e., conventional HMMs automatically impose geometric length distributions on their same-state regions, such as exon or intron lengths or blockade level durations. An HMM-with-Duration (HMMwD) is an HMM where true, or a much more complete, knowledge of the length distributions on same-state regions is incorporated into the model [6]. Here we describe a novel HMMwD where the non-geometric length distribution information is incorporated via dwell-time dependent transition probabilities (for transitions of state to self) [4]. New experimental results are shown, and compared to an exact HMMwD (described in [7]).

Part of the novelty of the new "cellular" HMMwD that is proposed is that it is defined at the cell-level in its dynamic programming table construction, much like the conventional HMM, with one column's computation only dependent on information held in the prior column (in an overall table computation involving a single pass through the table). Our HMMwD can be defined for either the Viterbi or the Forward/Backward algorithms (see Methods). This is convenient because we have a method for distributed HMM processing based on such table computations (paper in preparation), that takes advantage of the basic, underlying Markov assumption to do distributed processing with simplicity of the "chunk" distributed processing that we use for the SVM training [8]. That method is shown to work very effectively for the Viterbi algorithm (similar distribution methods for the Forward/Backward distribution algorithm are also discussed there). The end-result of all of this is that very sophisticated feature extraction tools can be brought to bear for real-time pattern recognition informed operation.

Real-time control of a nanopore detector, based on live, streaming measurements and sufficiently fast pattern recognition identification of any blockading ("captured") analyte, holds great promise for single molecule experiments. Real-time *sampling *control of a nanopore detector, alone, can boost nanopore detector sampling productivity by orders of magnitude, depending on the mix of desirable signal classes vs. undesirable in the data being analyzed. An example of such an experiment is the focus of the proof-of-principle experiment performed here. If there is a 1 to 100 ratio of desirable to undesirable, for example, then one obtains desirable signal sampling only about 1% of the time with a passive sampling system. With pattern recognition informed sampling this can potentially be changed to desirable signal sampling almost 99% of the time. In a real-time setting the challenge is to perform the HMM feature extraction sufficiently quickly (whereas the SVM is trained off-line, so operates very quickly on-line). In this work we show that this can be accomplished with pattern recognition used to identify DNA molecules within the first few hundred milliseconds of their blockade of the detector channel.

We establish a real-time experimental setup for Patter Recognition Informed (PRI) sampling control via integration of LabWindows automation capabilities with our "in-house" Channel Current Cheminformatics (CCC) methods (see Methods). Data acquired with LabWindows is passed to a network of CCC software clients, on a streaming real time basis, for analysis and classification. The classification results are then quickly returned to the LabWindows automation software for experimental feedback control. Further details of a real-time test of PRI sampling is described in the Results. The classification inherits the 99.9% accuracy of the non real-time implementation (established in prior work [1]) as nothing has changed in regards to the features extracted and the classifier used.

To make these Channel Current Cheminformatics and Machine Learning tools available to fellow researchers, we are developing web-accessible machine-learning tools. Using hidden Markov model (HMM) processing, and finite state automata (FSAs), we are able to extract robust features and obtain very accurate support vector machine (SVM) classification results. The Machine Learning web-interfaces described here are for both machine learning experts and non-experts, particularly biologists and biophysicists/biochemists. For non-experts, default values are specified on the key parameters.

Web-accessible tools for HMM-based feature extraction and SVM classification are accessible at . Examples of the Web interfaces are shown in the Results. The web tools can help in identification of blockade levels, the level transition and lifetime characteristics, and the fast blockade "spike" characteristics. The SVM classification is of general use for any kind of classification problem, and a number of novel kernels and novel implementations are employed. SVM-based clustering is also implemented in a novel way to yield a non-parametric clustering approach, which is used to cluster signals into multiple classes (particularly important for complex multi-orientation data-sampling situations such as with an antibody).

## Background

### Nanopore detector

Single biomolecules, and the ends of biopolymers such as DNA, have been examined in solution with nanometer-scale precision using nanopore blockade detection [1-3,9-11]. In early studies [2], it was found that complete base-pair dissociations of dsDNA to ssDNA, "melting", could be observed for sufficiently short DNA hairpins. In later work [1-3,9], the nanopore detector attained Angstrom resolution and was used to "read" the ends of dsDNA molecules, and was operated as a chemical biosensor. In [9-13], the nanopore detector was used to observe the conformational kinetics of the end regions of individual DNA hairpins (see Fig. 1, Lower Panel).

The α-hemolysin (α-HL) channel, a protein heptamer formed by seven identical 33 kD protein molecules secreted by *Staphylococcus aureus*, is used as the channel in the nanopore device due to its stable conformation (in the strongly favored heptamer formation, which has minimal gating) and its overall geometry (see Fig. 1, Lower Panel). DNA and RNA interaction with the α-hemolysin channel during translocation is non-negligible (but not too strong either, i.e., it is not such that the molecule "gets stuck"). Although dsDNA is too large to translocate, about ten base-pairs at one end can still be drawn into the large *cis*-side vestibule. This permits very sensitive experiments since the ends of "captured" dsDNA molecules can be observed for extensive periods of time to resolve features, allowing highly accurate classification of the captured end of dsDNA molecules [1-3,9-13]. This is a very brief and limited synopsis of the Nanopore Detector background relevant to this paper. For other references on Nanopore Detectors see the review of Nanopore Detectors presented in [14]; early work involving alpha-Hemolysin Nanopore Detectors can be found in [1-3,9-11,15-25]; rapidly growing research endeavors on Nanopore Detectors based on solid-state and other synthetic platforms can be found in [26-36].

### Cheminformatics

**Figure 2:**
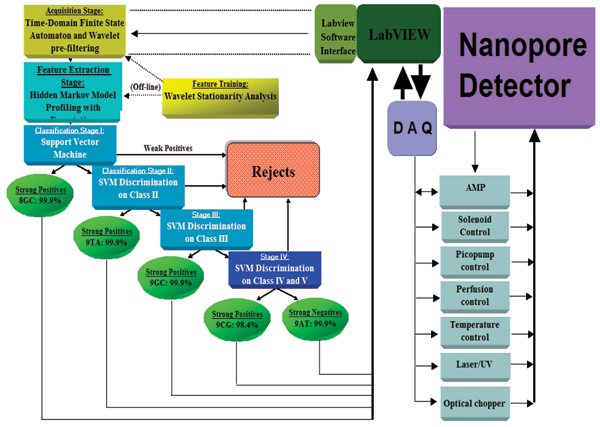
**Nanopore Cheminformatics and Control Architecture**. LabWindows Server now used. Data sent to cluster of Linux Clients via TCP/IP channel. Linux clients run expensive HMM analysis as distributed processes (similarly for off-line SVM training). The sample classification is used by the Server to provide feedback to the nanopore apparatus to increase the effective sampling time on the molecules of interest (this can boost nanopore detector productivity by magnitudes).

**Figure 3:**
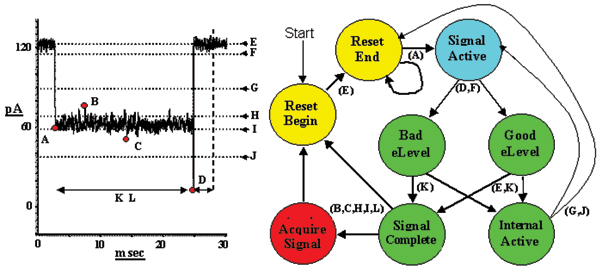
Time domain Finite State Automaton (τFSA) for detection/screening of molecular capture events at the nanopore channel site. All signal blockades are normalized to the average value of the baseline for the 20.48 ms prior to onset of blockade.

The pattern recognition informed (PRI) signal processing architecture builds and "closes the loop" on the prototype architecture presented in [1] (see Fig. 2). The signal processing architecture is used to perform a preliminary test of PRI sampling control (see Results). The processing is designed to rapidly extract useful information from noisy blockade signals using feature extraction protocols, wavelet analysis, Hidden Markov Models (HMMs) and Support Vector Machines (SVMs). For blockade signal acquisition and simple, time-domain feature-extraction, a time-domain Finite State Automaton (τFSA) approach is used [37] that is based on tuning a variety of threshold parameters (see Fig. 3 and [1] for full description of the model). A generic HMM is then used to characterize current blockades by identifying a sequence of sub-blockades as a sequence of state emissions [1,9-11]. The parameters of the generic-HMM can then be estimated using a method called Expectation/Maximization, or 'EM" [5], to effect de-noising. The HMM method with EM is part of the standard implementation used in what follows. Classification of feature vectors obtained by the HMM for each individual blockade event is then performed using SVMs.

### EVA projection

**Figure 4:**
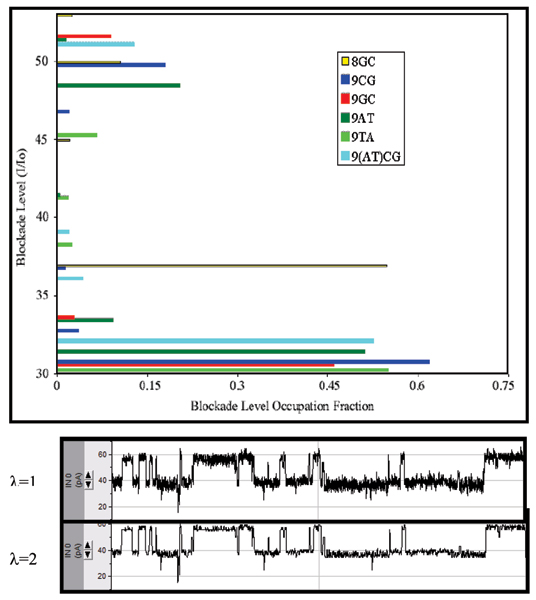
**HMM/EM Viterbi-path level occupation feature extraction**. Strong EVA projection (effects of λ = 2 shown) is employed to project the data onto dominant levels, a Viterbi path Histogram then shows the barcode "fingerprints" of the different molecular species. The labels are for the DNA hairpins examined in [1], and since then have been used as controls.

**Figure 5:**
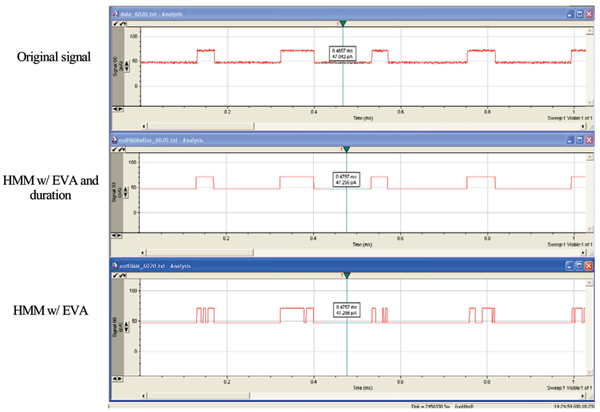
**Results using synthetic ~Poisson data**. Synthetic data with Poisson distributed length statistics is shown in the upper trace. Emission broadening is introduced with an emission variance amplification factor of 4.5. This effectively broadens the noise band (thickness) seen in the upper trace by a factor of 4.5, which leads to a blurring between the upper and lower levels of blockade since the noise bands now overlap (i.e., here we purposefully over-project to lead to the typical toggling cross-over instability shown in the bottom trace). The middle trace shows the clean, highly accurate Viterbi parsing into the appropriate levels that is obtained with use of the HMM-with-Duration implementation. The lower trace shows the Viterbi parse with a simple HMM, that is uninformed about the underlying length distributions, thus giving rise to a Viterbi traceback parse that fails to penalize unlikely, very short duration, blockade events (seen as the unstable, rapid level-projection toggles).

The HMM method is based on a stationary set of emission and transition probabilities. Emission broadening, via amplification of the emission state variances, is a filtering heuristic that leads to level-projection that strongly preserves transition times between major levels (see Fig. 4, and [4], and Methods for further details). This approach does not require the user to define the number of levels (classes), which is a major advantage compared to existing tools that require the user to determine the levels (classes) and perform a state projection. This allows kinetic features to be extracted with a "simple" FSA (Finite State Automaton) that requires minimal tuning. Figure 5 (reproduced from [4]) shows the benefits of EVA filtering.

### Preliminary HMM with Duration and EVA for channel current signal analysis

One important application of the HMM-with-duration method used in [4] includes kinetic feature extraction from EVA projected channel current data (the HMM-with-Duration is shown to offer a critical stabilizing capability in an example in [4]). The EVA-projected/HMMwD processing offers a hands-off (minimal tuning) method for extracting the mean dwell times for various blockade states (the core kinetic information).

The HMM-with-Duration implementation, described in [4], has been tested in terms of its performance at parsing synthetic blockade signals (see Fig. 5). The synthetic data ranges over an exhaustive set of possibilities for thorough testing of the HMM-with-Duration. The synthetic data used in [4] was designed to have two levels with lifetime in each level determined by a governing distribution (Poisson and Gaussian distributions with a range of mean values were considered). The HMM here was performed with 0 EM iterations.

### SVM classification used in PRI sampling selection

**Figure 6:**
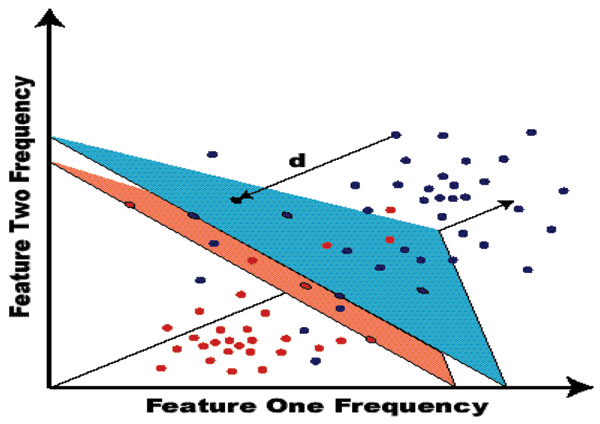
SVM: Hyperplane separability with a Margin (thickness). Support vectors consist of both the blue and red points occurring on the blue and red margin surfaces, respectively. Unlike HMM-based classification, the SVM-classification provides built-in confidence levels as part of the classification output.

Support Vector Machines (SVMs) are variational-calculus based methods that are constrained to have structural risk minimization (SRM) such that they provide noise tolerant solutions for pattern recognition [38,39]. Simply put, an SVM determines a hyperplane that optimally separates one class from another (see Fig. 6). Once learned, the hyperplane allows data to be classified according to the region (separated by the hyperplane) in which it resides. Currently there are two approaches to implementing *multiclass *SVMs. One arranges several *binary *classifiers as a decision tree such that they perform a multi-class decision-making function (SVM-external classification – an example of this is the architecture used here, see Fig. 2). The second approach involves solving a single optimization problem corresponding to the entire data set (with multiple hyperplanes), with multi-class discriminator optimization performed internally. The SVM-internal approach, when it is stable and properly generalizable (an active area of research), is preferred (see Results for interface), since a tuning over Decision tree topologies and weightings is avoided [40]. The on-line discriminatory speed of a trained SVM is simply that of evaluating an inner product, so it's operational constraint on the PRI feedback endeavor is negligible compared to that of the HMM feature extraction stage. For this reason, there is little discussion of SVMs in this paper, even though SVMs comprise much of the complexity of the HMM/SVM PRI feedback system.

### Generalized HMMs

The work by Johnson in [41] is an excellent source of references on generalized HMMs (but entirely focused on speech applications). The approaches to HMM duration described in [41] are broken into three categories: 1) Hidden semi-Markov models (HSMMs), 2) variable transition HMMs (VTHMMs), and 3) standard HMMs with more states, etc. The current work thus would fall into the VTHMM category. In [41] they assert that VTHMM methods are essentially variations on Ferguson's [42] explicit duration HMM (EDHMM), placed in the HSMM category, yet acknowledge the differences in computational complexity. In [43], Levinson gives estimates of transitions and emissions for state durations modeled by gamma distributions, but no explicit method provided or used to decode actual signals. In [44], Ramesh et al estimate the duration dependent state transitions, A_ij_(d), in general rather than assume, as in the current work, that A_ij_(d) = C_ij _for i ≠ j, but do not offer any performance analysis per se of the Viterbi decoding algorithm. Also, [44] provides insight into the work by Ferguson [41] in that, the expansion state HMM (ESHMM) in [41] uses multiple expansion states in order to finitely approximate the duration of a given state of the original system. The current work uses only one model state for each known physical state, and uses time dependent self-transition probabilities to capture state duration information.

In [45], Mitchell et al model duration explicitly using expansion substates, rather than an explicitly time dependent self-transition probability as in the current work. It also manages computational complexity via parallel platform architecture. The paper listed as [46], is the only prior work that is found to model the self-transitions using the cumulative duration in the form A_ii_(d_i_) = 1 - P(d_i_), and this was only in a much more restricted sense than that described here. The authors there considered only a two-state system for which the "splitting" in probabilities upon exiting one state is trivial. In [46], Vaseghi computes the duration dependent state transitions directly from the Viterbi output, thus avoiding the costly forward-backward computation and this is similar the methods employed here for general multi-state HMMs. This is useful in situations where the exit transitions are not already known and must be estimated from the data. Otherwise, like most of the references included here, though it can apply specifically to the current work, it involves algorithms that are more generic and computationally complex and not the clear, extremely fast, and simple implementation described here.

As in most other works cited here, Sin et al. [47] involves more generic computations and hence more computational complexity, though in the examples provided avoids the complexity of multiple exit transitions from any given state by restricting the actual computations performed to only left-to-right type models, where there is only one exit transition per state. (Future work might be to use the framework in [47] to confirm the choice of splitting factor for multiple exit transitions, see Discussion.) Park et al [48] also performs analysis using only left-to-right models, where the splitting factor for multiple exit transitions is not an issue. Though emissions for a given state in cheminformatic data are not expected to be time-dependent – other than noise component, future work would be to use the framework in [48] to confirm stationary emissions. Finally, in Yoma et al. [49] the analysis is restricted again to the typical left-to-right models of speech recognition, where considerations of splitting factor for exit transitions are trivial.

## Results

### HMM with Duration experimental tests

**Figure 7:**
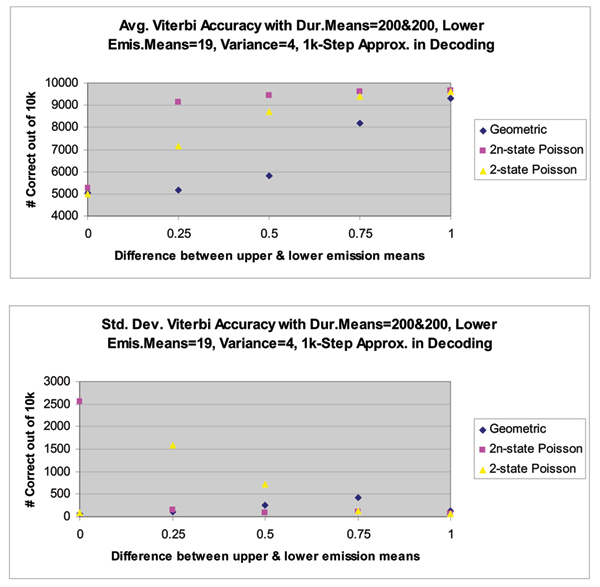
Average and Standard Deviation of Viterbi Decoding Accuracy over 10 different trials (instances) of 10 k-length synthetic 2-level signal data, where both levels have identical Poisson duration but the separation between the 2 levels varies. From top to bottom, the Viterbi response improves as the number of steps increases in the decoding HMM's approximation of the 1 k-step generating HMM's Poisson durations. From left to right in each plot, the Viterbi response improves as the separation of the 2 levels (emission means) increases. Result of 1 k steps used in the Viterbi decoding HMM's approximation of the 1 k-step generating HMM's Poisson durations.

Results for our new, implicit HMMwD, are presented in Figure 7 and in the figures in Additional Files 1–3, along with the comparative results from an explicit HMMwD (for a two state system) that is used for comparative analysis (a detailed analysis with the explicit HMMwD is given in [7]). The explicit two-state HMMwD has 2*n *states, where *n *is the number of dwell bins in the quantization of the dwell-time distribution. The computations that are needed scale quadratically in *n*. So, for *n *= 1000, the explicit HMMwD can take 1,000,000-times longer to compute than the HMMwD described here (or the conventional HMM). Performance of the new HMMwD is given in comparison to the conventional HMM is also shown in Figure 7 and in the figures in Supplemental Files 1–3. Specifically, the figures in Additional File 1–3 show the Average and Standard Deviation of Viterbi Decoding Accuracy over 10 different trials (instances) of 10 k-length synthetic 2-level signal data, where both levels have identical Poisson duration but the separation between the 2 levels varies. From top to bottom, the Viterbi response improves as the number of steps increases in the decoding HMM's approximation of the 1 k-step generating HMM's Poisson durations. From left to right in each plot, the Viterbi response improves as the separation of the 2 levels (emission means) increases. In the figure in Additional File 1 are shown the results with only 1 step used in the Viterbi decoding HMM's approximation of the 1 k-step generating HMM's Poisson durations. In the figure in Additional File 2 are shown the results with 10 steps used in the Viterbi decoding HMM's approximation of the 1 k-step generating HMM's Poisson durations. In the figure in Additional File 3 are shown the results with 100 steps used in the Viterbi decoding HMM's approximation of the 1 k-step generating HMM's Poisson durations. The simplicity of the binary signal case actually provides a challenging case for differentiating the performance of the HMMwD methods from the conventional HMM, so the preliminary results shown here show promise for the overall validity and utility of the method.

**Figure 8:**
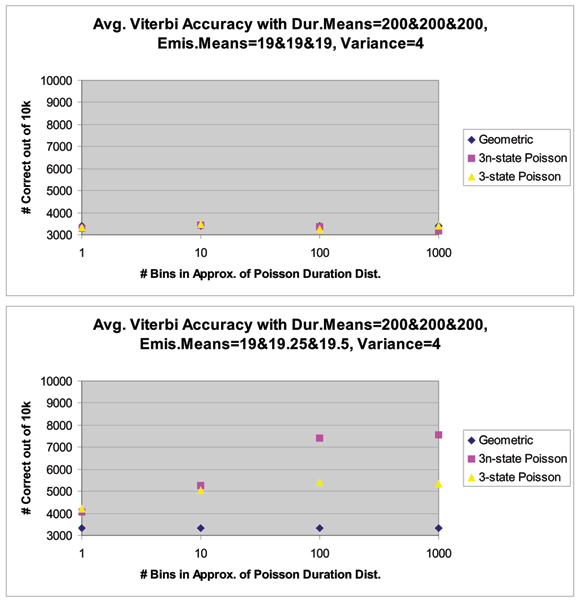
Average Viterbi Decoding Accuracy over 10 different trials (instances) of 10 k-length synthetic 3-level signal data, where all levels have identical Poisson duration but the separation (gaussian emission means) between the levels varies. The Viterbi decoding accuracy improves as the number of bins increases in the decoding HMM's approximation of the Poisson durations generated using a 1 k-bin length distribution representation in the generating HMM. From left to right in each plot, the Viterbi response improves as the separation of the 3 levels (emission means) increases. **Top**, decoding performance when all levels have identical attributes is random 3-way guessing, so the expected 3333 out of 10000 correct is observed in all cases. **Bottom**, decoding performance with distributions with means 19 (for geometric), 19.25 and 19.5 (with Poisson distributed dwell-times).

**Figure 9:**
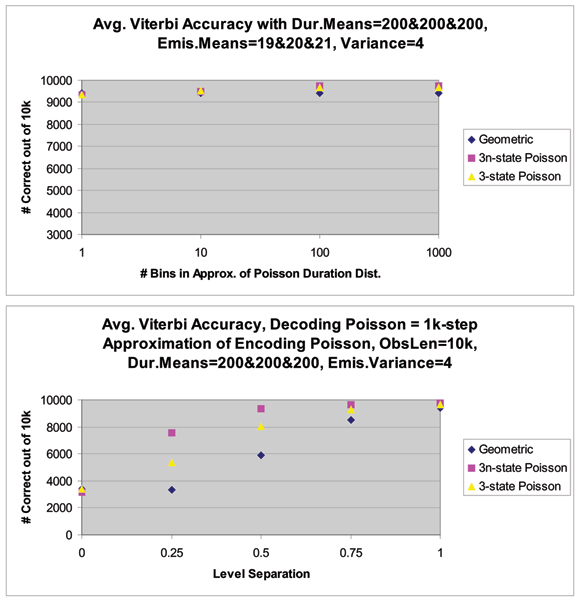
**Top**, decoding performance with distributions with means 19 (for geometric), 20 and 21 (with Poisson distributed dwell-times). **Bottom**, decoding performance with distributions with different mean separations, with a 1000-bin representation of the state dwell-time distribution. (See Fig. 11 caption for further details.)

The next example considered is that of a 3-state system, again, the strong performance of our new HMM-with-Duration method is demonstrated, see Figures 8 and 9 and the figure in Additional File 4. The figure in Additional File 4 shows the decoding performance with distributions with means 19 (for geometric), 19.5 and 20 (with Poisson distributed dwell-times) (in the Top panel), and in the Bottom Panel the figure shows decoding performance with distributions with means 19 (for geometric), 19.75 and 20.5 (with Poisson distributed dwell-times). As a challenging, preliminary, test case, the duration means for all the distributions are kept the same (at 200). (See Fig. 8 caption for further details.)

In another test, we compare the performance of our two-state HMMwD to explicit HMMwD and conventional HMM (see Tables 1 and 2), with different size data generation and length distribution representation. The comparative scores shown are not optimised for use of the internal HMMwD, but are meant to explore the validity of the model and the behaviour of the accuracy of the different methods – which is consistently ordered as conventional HMM good, implicit HMMwD better, explicit HMMwD best (but the latter at much great computational cost). The results clearly demonstrate the superior performance of the HMM-with-duration over its simpler HMM without Duration formulation. With use of the EVA-projection method this affords a robust means to obtain kinetic feature extraction. The HMM with duration is, thus, critical for accurate kinetic feature extraction, via a pairing of the HMM-with-Duration stabilization with EVA-projection.

**Table 1:** For all row entries the average dwell time of both the upper and lower signal levels increases proportionately with bin-count.

Bin #	2-state Geometric HMM	2n-state Explicit HMMwD	2-state Implicit HMMwD
10	743876	832514	756765
100	951916	960272	953151
1000	995388	995408	995381

Thus the resolution of the duration distribution for both the signal-generating and the signal-decoding HMM's are identical for all row entries. Upper and lower mean durations are .2 and .6 times that of the bin count, respectively. Upper and lower mean emissions are 50.0 and 45.0, respectively, whereas the emission variance for both upper and lower is 20.0. In other tests (see Fig. 7 and figures in the Additional Files), the decoding duration distribution is an increasingly refined step function approximation of the generating duration distribution. For the data shown in this table, both upper and lower dwell-time distributions are 2-component beta-mixtures that are monotonically decreasing. The numbers shown represent the accuracy of Viterbi decoding out of an input signal 1 M samples in length.

**Table 2:** As with Table 1, for all row entries the average dwell time of both the upper and lower signal levels increases proportionately with bin-count.

Bin #	2-state Geometric HMM	2n-state Explicit HMMwD	2-state Implicit HMMwD
10	720050	795723	741579
100	947062	957753	948505
1000	994978	995013	995005

Thus the resolution of the duration distribution for both the signal-generating and the signal-decoding HMM's are identical for all row entries. Upper and lower mean durations are .2 and .6 times that of the bin count, respectively. Upper and lower mean emissions are 50.0 and 45.0, respectively, whereas the emission variance for both upper and lower is 20.0. In other tests (see Fig. 7 and figures in the Additional Files), the decoding duration distribution is an increasingly refined step function approximation of the generating duration distribution. For the data shown in this table, both upper and lower durations are 3-component beta-mixtures with humps in the aggregate.

### Pattern recognition informed feedback via LabWindows automation

A blockade signal's stationary statistics, or phases thereof, reveals information about the kinetics of the biopolymer resulting from interactions with surroundings, or from undergoing conformational changes. LabVIEW Automation software is used to help manage the feedback linkage between patch-clamp amplifier measurements and in-house cheminformatics software. This has been used to demonstrate molecular identification in the first 100 ms of capture, with return of classification information to the control of the amplifier – for voltage-controlled sample ejection if desired. Screen-captures of the interfaces are shown in the figures in Additional Files 5–7. Additional File 5 shows the Acquisition Server interface and LabWindows C development environment. Additional File 6 shows a real time 9at vs 9gc classification (with 9at identification indicated by the led being on). Additional File 7 shows a real time 9gc identification.

The LabWindows Server initiates the distributed CCC computations by sending data to a cluster of Linux Clients via a TCP/IP channel. The Linux clients run the expensive HMM analysis as distributed processes (similarly for off-line SVM training). The sample classification is then used by the Server to provide feedback to the nanopore apparatus to increase the effective sampling time on the molecules of interest (meant to boost nanopore detector productivity by magnitudes, as described in the Background).

### Channel current cheminformatics and Machine-Learning web interfaces

The tFSA/HMM-based channel current cheminformatics web-interfaces are as follows: (1), As the tFSA runs, signal regions are identified for processing by the HMM. (2), A 50-state generic-HMM (typically) is used to obtain feature vectors, to be used by a SVM, consisting of three components: (i) Blockade Histogram: blockade level occupation probabilities (stat's on Viterbi path); (ii) The Emission Variances; and (iii) Compression of Transition probabilities to weighted sum of transition profiles from two dominant levels (the UL and LL states) (one of the interfaces for this process is shown in the figure in Additional File 8). And (3), Kinetic features are then extracted (often with EVA-projected data from step 2). An SVM interface and all other software described is available via the webpage: . As with the external and internal multiclass SVM discriminator implementations, the strong performance of the binary SVM enables SVM-External as well as SVM-Internal approaches to clustering [40].

## Methods and discussion

### Conventional HMM

An HMM consists of 2 main computations (Durbin et al [5]):

1. Baum-Welch Iteration of recursively defined forward/backward probabilities (symbols x, states π)

f_*l*_(*i *+ 1) = P(x_1_...x_*i*+1_, π_*i*+1 _= *l*) = *e*_*l*_(x_*i*+1_)π_*k*_f_*k*_(*i*)*a*_*kl*_

b_*k*_(*i*) = P(x_*i*+1_...x_*L*_| π_*i *_= *k*) = π_*l*_*a*_*kl*_*e*_*l*_(x_*i*+1_)b_*l*_(*i *+ 1)

2. Viterbi Path Determination

v_*l*_(*i*) = *e*_*l*_(x_*i*_)max_*k*_(v_*k*_(*i *- 1)*a*_*kl*_)

ptr_*i*_(*l*) = argmax_*k*_(v_*k*_(*i *- 1)*a*_*kl*_)

In the HMMwD described here, each of the stationary transition probabilities a_*kl *_are replaced by a dwell-time dependent update factor. The forward/backward probabilities used in the standard HMM-EM algorithm occur when evaluating p(Z_0...L-1_) by breaking the sequence probability p(Z_0...L-1_) into two pieces via use of a single hidden variable treated as a Bayesian parameter: p(Z_0...L-1_) = Σ_k_p(Z_0...i_, s_i _= k)p(Z_i+1...L-1_, s_i _= k) = Σ_k_f_ki_b_ki_, where f_ki _= p(Z_0...i_, s_i _= k) and b_ki _= p(Z_i+1...L-1_, s_i _= k). Given stationarity, the state transition probabilities and the state probabilities at the ith observation satisfy the trivial relation p_qi _= Σ_k_a_kq_p_k(i-1)_, where p_qi _= p(S_i _= q), and p_q0 _= p(S = q), and the latter probabilities are the state priors. The trivial recursion relation that is implied can be thought of as an operator equation, with operation the product by a_kq _followed by summation (contraction) on the k index. The operator equation can be rewritten using an implied summation convention on repeated Greek-font indices (Einstein summation convention): p_q _= a_βq_p_β_. Transition-probabilities in a similar operator role, but now taking into consideration local sequence information via the emission probabilities, are found in recursively defined expressions for the forward variables, f_ki _= e_ki_(a_βk_f_β(i-1)_), and backward variables, b_ki _= a_kβ_e_β(i+1)_b_β(i+1)_. The recursive definitions on forward and backward variables permit efficient computation of observed sequence probabilities using dynamic programming tables. It is at this critical juncture that side information must mesh well with the states (column components in the table), i.e., in a manner like the emission or transition probabilities. Length information, for example, can be incorporated via length-distribution-biased transition probabilities (introduced in [4]), and that is what is experimentally validated done here.

### EVA projection

Using a standard implementation of a HMM with emissions probabilities parameterized by Gaussian distributions: emission_probabilities[i][k] = exp(-(k - i)*(k - i)/(2*variance)), where "i" and "k" are each a state where 0 <= i, k <= 49 in a 50 state system. To perform EVA, the variance is simply multiplied by a factor that essentially widens the gaussian distribution parameterized to best fit the emissions, and the equation simply becomes exp(-(k-i)*(k-i)/(2*variance*eva_factor)). The choice of this amplification factor is important. If too large of a factor is used, then the power signal will be altered to the point where the state transition information will be invalid. But for a sizable range of this parameter, HMM with EVA will remove the noise from the power signal while *strictly *maintaining the state transitions. In practice, any multiplicative factor between 2 and 10 works well.

After EVA-projection, a simple FSA can easily extract level duration information. Each level is identified by a simple threshold of blockade readings, typically one or two percent of baseline. Then, the HMM with EVA processed data is swept through with a small window to eliminate any remaining noise or spike artifacts that may distort actual level duration statistics. It is important to note that there must be a sufficient amount of data to support the level duration statistics. A small sample will simply not be representative of the true kinetic information. Further, it is important that the FSA is tuned properly in order to properly identify levels.

### HMM with Duration via cumulant transition probability

The transition probabilities for state 'e' to remain in state 'e', a "ee" transition can be computed as: Prob(ee | e_length _= L) = Prob(e_length _≥ L + 1)/Prob(e_length _≥ L). The transition probabilities out of state 'e' can have some subtleties, as shown in the following where the states are exon (e), intron (i), and junk (j). In this case, the transition probabilities governing the following transitions, (jj) -> (je), (ee) -> (ej), (ee) -> (ei), (ii) -> (ie) are computed as: Prob(ei | e_length _= L) = Prob(e_length _= L)/Prob(e_length _≥ L) × 40/(40 + 60) and Prob(ej | e_length _= L) = Prob(e_length _= L)/Prob(e_length _≥ L) × 60/(40 + 60), where the total number of (ej) transitions is 60 and the total number of (ei) transitions is 40. The pseudocode to track the critical length information, on a cellular basis in the dynamic programming table, goes as follows:

1. Maintain separate counters for the junk, exon and intron regions.

2. The counters are updated as follows:

a. The exon counter is set to 2 for a (je) - (ee) transition

b. The exon counter gets incremented by 1 for every (ee) - (ee) transition

Prob(e_length _≥ L + 1) is computed as: Prob(e_length _≥ L + 1) = 1 - ∑_i=1..L_Prob(e_length _= i). Hence we generate a list such that for each index k > 0, the value 1 - ∑_i=1..k_Prob(e_length _= i) is stored.

Simplifying from three state model, {e, i, j}, to a two-state model, {e, i}, for a moment: after n occurrences of state 'e', the 2 cases of update factor to handle are:

P(e_n+1_|ee..e_n_) = P(length e ≥ n+1)/P(length e ≥ n)

P(i|ee..e_n_) = P(length e = n)/P(length e ≥ n)

Similarly for n occurrences of state 'i', and there are no probability splitting ambiguities upon exiting state 'e' as there is only one state to exit to in the two-state system (and there are no differences in the Viterbi and Forward/Backward transition probability alterations).

Consider, as an example, a simple extension of our two-state notation to cover N + 1 states: {e, i^1^,...,i^N^}. Suppose we are interested in the probability of an 'i' after seeing a length 4 segment of e-states:

P(i|eeee) = 1 - P(e|eeee) = 1 - P(L_e _= 5)/P(L_e _= 4)

There are two types of transition rule that appear to result, one for Viterbi, with its **maximum **operation on paths, and one for Forward/Backward, with its **sum **operation. For the two-state case, N = 1, and these update rules involving "splitting" factors all become the same (see Results):

#### Viterbi update

(1) Difference splitting: p(i^k^|eeee) = [1 - P(L_e _= 5)/P(L_e _= 4)] * [1 + P(i^k^|e) - Avg_z_P(Z|e)]

(2) Ratio splitting: p(i^k^|eeee) = [1 - P(L_e _= 5)/P(L_e _= 4)] * P(i^k^|e)/[Avg_z_P(Z|e)]

In this situation we are not maintaining a sum rule on probabilities, here we are viewing each path through the table in a manner consistent with the maxprob evaluation.

#### Forward/Backward update

(1) Difference splitting: p(i^k^|eeee) = [1 - P(L_e _= 5)/P(L_e _= 4)] * [1 + P(i^k^|e) - Avg_z_P(Z|e)]/N

(2) Ratio splitting: p(i^k^|eeee) = [1 - P(L_e _= 5)/P(L_e _= 4)] * P(i^k^|e)/[N*Avg_z_P(Z|e)]

The equation above with the factor [1 + P(i^k^|e) - Avg_z_P(Z|e)]/N provides a suitable "splitting factor", as i^k ^and 'e' probabilities sum to one, remain positive, and have other nice properties. The splitting factor is not unique, however, as case (2) makes clear.

It is important to note that we appear to have some freedom on splittings of probabilities upon exiting a state (when we are using the length distribution cumulants to describe transition probabilities, etc.). This is merely an associated effect of that length distribution incorporation – now upon exiting that length distribution our main factor is P(i^k^|some prior length of e), that factor is blind to the appropriate splittings amongst the "not e" states, and we must incorporate another factor to deal with the probability splitting – in the case of forward/backward, this is chosen to obey a prob sum to 1 on all cases, on Viterbi this must maintain each path's probability with proper weighting with respect to the others (consistent with the max-path operation).

### Real-time processing hardware/software setup

The server was able to concurrently perform the following using a single 1.5 GHz processor with no hyper-threading:

1. Acquire data from the DAQ buffer at 50 KHz sample rate.

2. Update the server GUI screen with the acquired data – though a 10× data decimation was required in order to avoid irrevocable delays in reading from the DAQ buffer

3. Perform tFSA logic to screen for signals resulting from molecular capture events at the nanopore channel

4. Send capture-signals as long as 100 ms in duration at a rate 10 per second to a waiting 1.5 GHz processor, Linux-based TCP/IP client for HMM-based feature extraction

5. Receive extracted HMM features from the TCP/IP client

6. Compute the classification of the HMM features with tolerance via a binary SVM previously trained on 9gc and 9at bphp signals.

7. According to the user's preset preference, issue a control signal to the DAQ resulting in ejection of the undesirable molecule so determined from the nanopore channel site.

## Competing interests

The authors declare that they have no competing interests.

## Authors' contributions

The paper was written by SWH. The HMM with Duration method was developed by SWH and implemented by CB and SWH. The PRI feedback experimental approach was implemented by CB under the supervision of SWH. The dataruns for both the HMM-with-Duration results and the PRI-feedback results were performed by CB.

## Supplementary Material

Additional file 1 Further Results for with the new  HMM-with-duration formalism (see Fig. 7), with only 1 step used in the Viterbi decoding HMM’s approximation of the 1k-step generating HMM’s Poisson durations. Click here for file

Additional file 2 Further Results for with the new  HMM-with-duration formalism (see Fig. 7), with 10 steps used in the Viterbi decoding HMM’s approximation of the 1k-step generating HMM’s Poisson durations. Click here for file

Additional file 3 Further Results for with the new  HMM-with-duration formalism (see Fig. 7), with 100 steps used in the Viterbi decoding HMM’s approximation of the 1k-step generating HMM’s Poisson durations. Click here for file

Additional file 4 Results on a 3-state system, showing the strong performance of the new HMM-with-Duration method is demonstrated (see Figures 8 and 9 for other results on this system). Click here for file

Additional file 5 The Acquisition Server interface and LabWindows C development environment. Click here for file

Additional file 6 A real time 9AT vs 9GC DNA hairpin classification (with 9AT identification indicated by the LED light flashing on). Click here for file

Additional file 7 An image showing a real time 9GC identification event. Click here for file

Additional file 8 A 50-state generic-HMM (typically) is used to obtain feature vectors,  a web-interfaces for this tool is shown. Click here for file

## References

[B1] Winters-Hilt S, Vercoutere W, DeGuzman VS, Deamer DW, Akeson M, Haussler D (2003). Highly Accurate Classification of Watson-Crick Basepairs on Termini of Single DNA Molecules. Biophys J.

[B2] Vercoutere W, Winters-Hilt S, Olsen H, Deamer DW, Haussler D, Akeson M (2001). Rapid discrimination among individual DNA hairpin molecules at single-nucleotide resolution using an ion channel. Nat Biotechnol.

[B3] Vercoutere W, Winters-Hilt S, DeGuzman VS, Deamer D, Ridino S, Rogers JT, Olsen HE, Marziali A, Akeson M (2003). Discrimination Among Individual Watson-Crick Base-Pairs at the Termini of Single DNA Hairpin Molecules. Nucl Acids Res.

[B4] Winters-Hilt S Hidden Markov Model Variants and their Application. BMC Bioinformatics.

[B5] Durbin R (1998). Biological sequence analysis: probalistic models of proteins and nucleic acids.

[B6] Koski T (2001). Hidden Markov Models for Bioinformatics.

[B7] Churbanov A, Baribault C, Winters-Hilt S (2007). Duration learning for nanopore ionic flow blockade analysis. BMC Bioinformatics.

[B8] Osuna E, Freund R, Girosi F, Principe J, Gile L, Morgan N, Wilson E (1997). An improved training algorithm for support vector machines. Neural Networks for Signal Processing VII.

[B9] Winters-Hilt S Nanopore detection using channel current cheminformatics. SPIE Second International Symposium on Fluctuations and Noise, 25–28 May, 2004.

[B10] Winters-Hilt S, Akeson M (2004). Nanopore cheminformatics. DNA and Cell Biology.

[B11] Winters-Hilt S (2003). Highly Accurate Real-Time Classification of Channel-Captured DNA Termini. Third International Conference on Unsolved Problems of Noise and Fluctuations in Physics, Biology, and High Technology.

[B12] Winters-Hilt S, Landry M, Akeson M, Tanase M, Amin I, Coombs A, Morales E, Millet J, Baribault C, Sendamangalam S Cheminformatics Methods for Novel Nanopore analysis of HIV DNA termini. BMC Bioinformatics.

[B13] Winters-Hilt S, Davis A, Amin I, Morales E (2007). Nanopore current transduction analysis of protein binding to non-terminal and terminal DNA regions: analysis of transcription factor binding, retroviral DNA terminus dynamics, and retroviral integrase-DNA binding. BMC Bioinformatics.

[B14] Ken Healy K (2006). Nanopore-Based DNA Analysis. PhD Thesis.

[B15] Bezrukov SM, Vodyanoy I, Parsegian VA (1994). Counting polymers moving through a single ion channel. Nature.

[B16] Bezrukov SM (2000). Ion Channels as Molecular Coulter Counters to Probe Metabolite Transport. J Membr Biol.

[B17] Kasianowicz JJ, Brandin E, Branton D, Deamer DW (1996). Characterization of Individual Polynucleotide Molecules Using a Membrane Channel. Proc Natl Acad Sci USA.

[B18] Akeson M, Branton D, Kasianowicz JJ, Brandin E, Deamer DW (1999). Microsecond time-scale discrimination among polycytidylic acid, polyadenylic acid, and polyuridylic acid as homopolymers or as segments within single RNA molecules. Biophys J.

[B19] Meller A, Nivon L, Brandin E, Golovchenko J, Branton D (2000). Rapid nanopore discrimination between single polynucleotide molecules. Proc Natl Acad Sci USA.

[B20] Meller A, Branton D (2002). Single molecule measurements of DNA transport through a nanopore. Electrophoresis.

[B21] Henrickson SE, Misakian M, Robertson B, Kasianowicz JJ (2000). Driven DNA transport into an asymmetric nanometer-scale pore. Phys Rev Lett.

[B22] Meller A, Nivon L, Branton D (2001). Voltage-driven DNA translocations through a nanopore. Phys Rev Lett.

[B23] Howorka S, Cheley S, Bayley H (2001). Sequence-specific detection of individual DNA strands using engineered nanopores. Nat Biotechnol.

[B24] Deamer DW, Akeson M (2000). Nanopores and nucleic acids: prospects for ultrarapid sequencing. Trends Biotechnol.

[B25] Branton D, Meller A, Kasianowicz JJ (2002). Using nanopores to discriminate between single molecules of DNA. Structure and Dynamics of Confined Polymers.

[B26] Heins E, Albertorio F, Yang T, Siwy Z, Baker L, Cheley S, Bayley HP, Cremer PS, Martin CR (2005). Development of a supported lipid bilayer on porous polymeric support. presented at the 49th Biophysical Society Meeting, Long Beach, CA, USA.

[B27] Li J, Stein D, M C, Branton D, Aziz M, Golovchenko J (2001). Ion beam sculpting on the nanoscale. Nature.

[B28] Stein D, Li J, Golovchenko JA (2002). Ion-beam sculpting time scales. Phys Rev Lett.

[B29] Stein DM, McMullan CJ, Li J, Golovchenko JA (2004). Feedback-controlled ion beam sculpting apparatus. Rev Sci Instrum.

[B30] Mitsui T, Stein D, Kim Y-R, Hoogerheide D, Golovchenko JA (2006). Nanoscale volcanoes: accretion of matter at ionsculpted nanopores. Phys Rev Lett.

[B31] Chen P, Mitsui T, Farmer DB, Golovchenko J, Gordon RG, Branton D (2004). Atomic layer deposition to fine-tune the surface properties and diameters of fabricated nanopores. Nano Lett.

[B32] Storm AJ, Chen JH, Ling XS, Zandbergen HW, Dekker C (2003). Fabrication of solid-state nanopores with single-nanometre precision. Nat Mater.

[B33] Heng J, Dimitrov V, Grinkova Y, Ho C, Kim T, Muller D, Sligar S, Sorsch T, Twesten R, Timp R, Timp G The detection of DNA using a silicon nanopore. Proc IEDM.

[B34] Siwy Z, Dobrev D, Neumann R, Trautmann C, Voss K (2003). Electro-responsive asymmetric nanopores in polyimide with stable ion-current signal. Appl Phys A.

[B35] Mara A, Siwy Z, Trautmann C, Wan J, Kamme F (2004). An asymmetric polymer nanopore for single molecule detection. Nano Lett.

[B36] Apel PY, Korchev YE, Siwy Z, Spohr R, Yoshida M (2001). Diode-like single-ion track membrane prepared by electrostopping. Nucl Instrum Methods Phys Res, Sect B.

[B37] Cormen TH, Leiserson CE, Rivest RL (1989). Introduction to Algorithms.

[B38] Vapnik VN (1998). The Nature of Statistical Learning Theory.

[B39] Burges CJC (1998). A tutorial on support vector machines for pattern recognition. Data Min Knowl Discov.

[B40] Winters-Hilt S, Yelundur A, McChesney C, Landry M Support Vector Machine Implementations for Classification & Clustering. BMC Bioinformatics.

[B41] Johnson M (2005). Capacity and Complexity of HMM Duration Modeling Techniques. IEEE Signal Processing Letters.

[B42] Ferguson J (1980). Variable duration models for speech. Proc Symp App Hidden Markov Models Text Speech.

[B43] Levinson S (1986). Continuously variable duration hidden Markov models for speech analysis. Proc Int Conf Acoust, Speech, Signal Process.

[B44] Ramesh P, Wilpon J (1992). Modeling state durations in hidden Markov models for automatic speech recognition. Proc Int Conf Acoust, Speech, Signal Process.

[B45] Mitchell C, Harper M, Jamieson L, Helzerman R (1995). A Parallel Implementation of a Hidden Markov Model with Duration Modeling for Speech Recognition. Dig Sig Proc.

[B46] Vaseghi S (1995). State duration modeling in hidden Markov models. Signal Process.

[B47] Sin B, Kim J (1995). Nonstationary hidden Markov model. Signal Process.

[B48] YK Park, Un CK, Kwon OW (1996). Modeling acoustic transitions in speech by modified hidden Markov models with state duration and state duration-dependent observation probabilities. IEEE Trans Speech Audio Process.

[B49] Yoma N, McInnes F, Jack M, Stump S, Ling L (2001). On including temporal constraints in viterbi alignment for speech recognition in noise. IEEE Trans Speech Audio Process.

