# Supplementary figures and images for: A novel, fast, HMM-with-Duration implementation – for application with a new, pattern recognition informed, nanopore detector
Source: BMC Bioinformatics. 2007 Nov 1;8(Suppl 7):S19. doi: 10.1186/1471-2105-8-S7-S19 (PMC2099487; doi:10.1186/1471-2105-8-S7-S19)

**
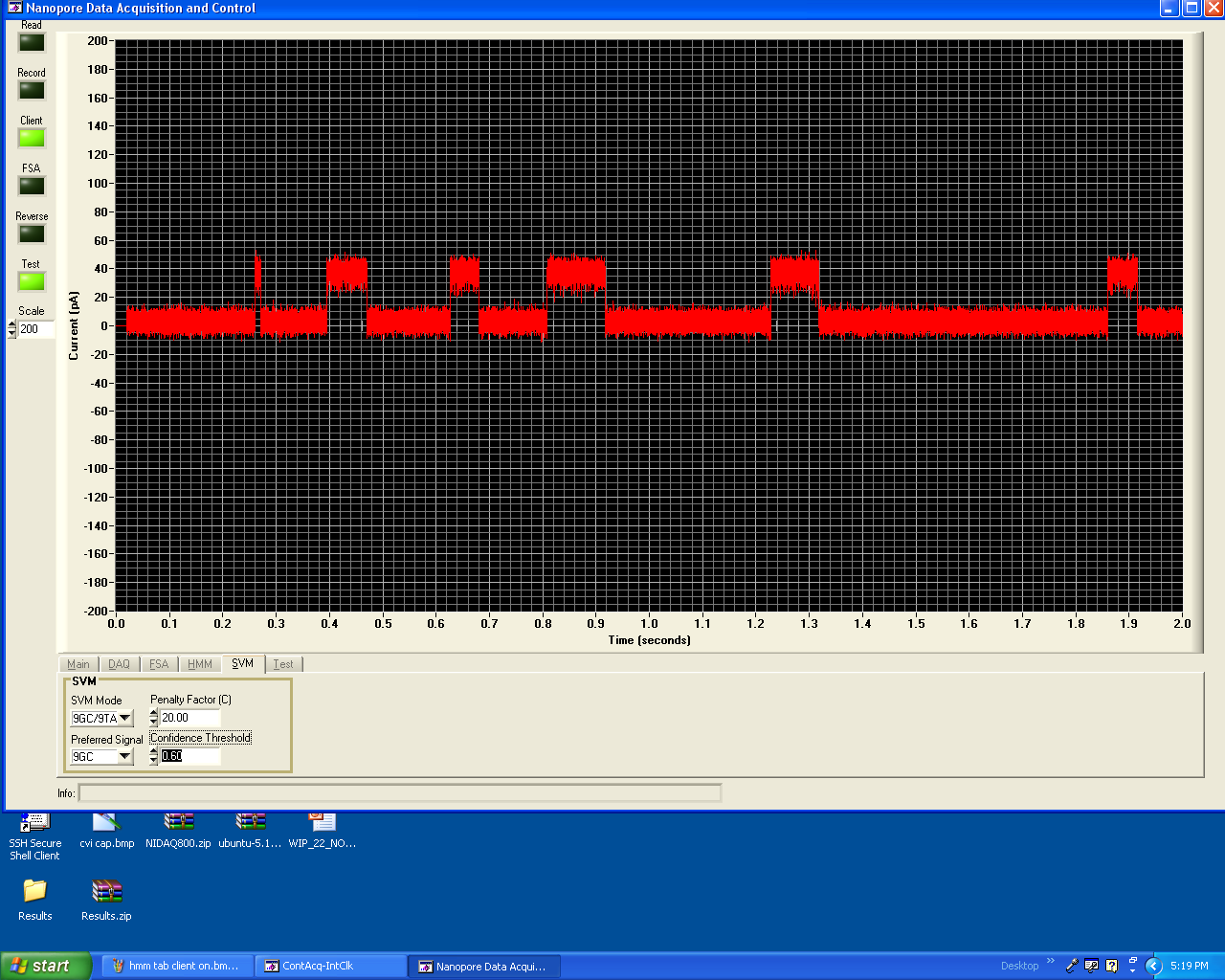
**

Supplement: Additional file 5 — The Acquisition Server interface and LabWindows C development environment. [file 1471-2105-8-S7-S19-S5.doc]

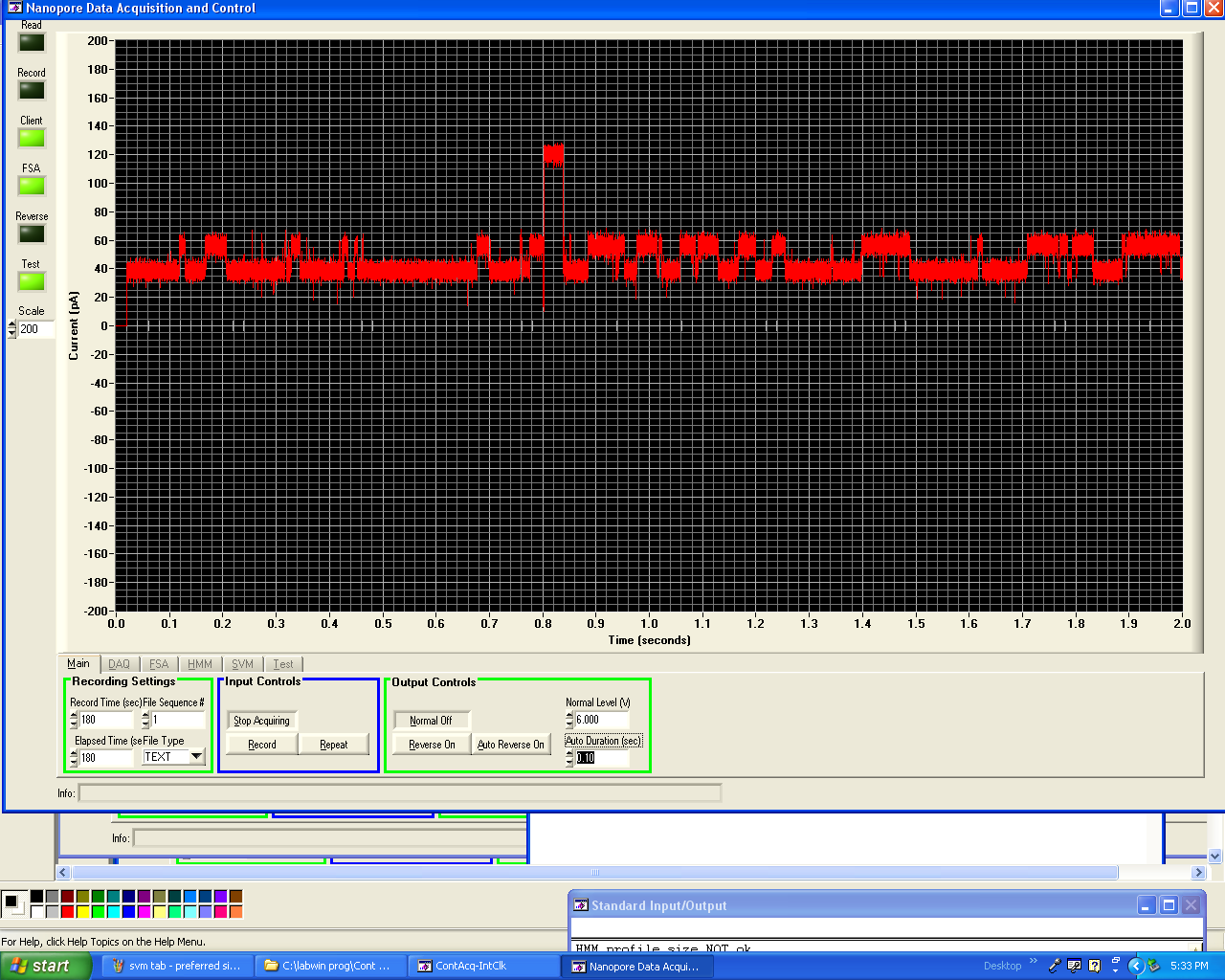

Supplement: Additional file 6 — A real time 9AT vs 9GC DNA hairpin classification (with 9AT identification indicated by the LED light flashing on). [file 1471-2105-8-S7-S19-S6.doc]

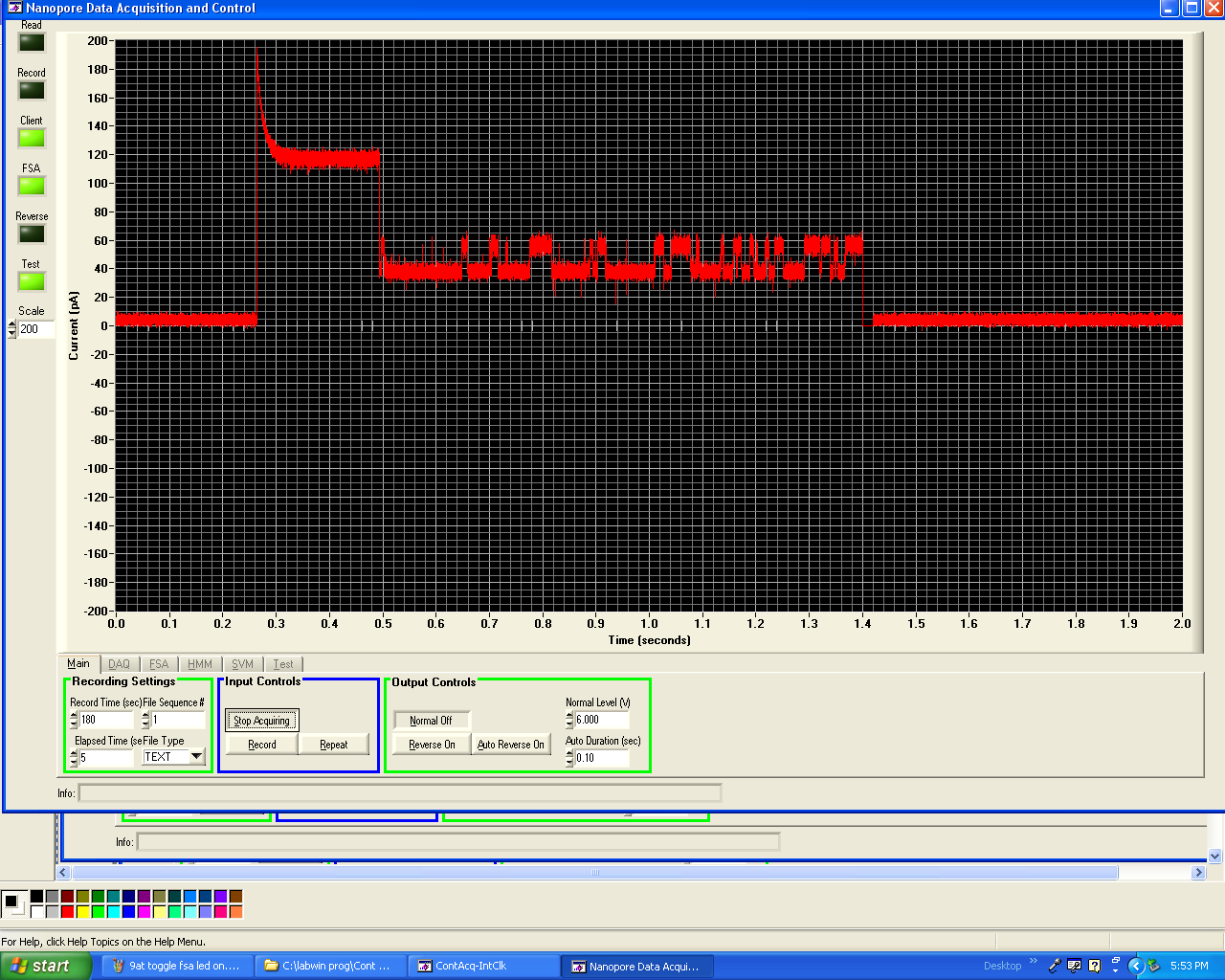

Supplement: Additional file 7 — An image showing a real time 9GC identification event. [file 1471-2105-8-S7-S19-S7.doc]
