# Supplementary material for: A novel, fast, HMM-with-Duration implementation – for application with a new, pattern recognition informed, nanopore detector
Source: BMC Bioinformatics. 2007 Nov 1;8(Suppl 7):S19. doi: 10.1186/1471-2105-8-S7-S19 (PMC2099487; doi:10.1186/1471-2105-8-S7-S19)
Supplement: Supplementary file 4 [file 1471-2105-8-S7-S19-S3.doc]

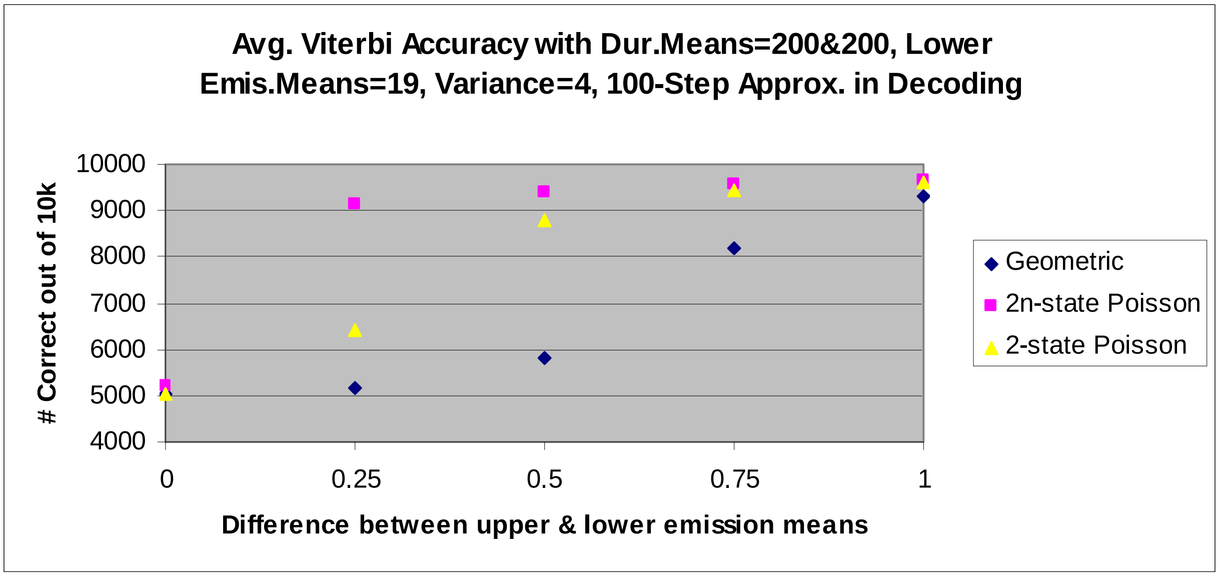
Avg. Viterbi Accuracy with Dur.Means=200&200, Lower Emis.Means=19, Variance=4, 100-Step Approx. in Decoding
Correct out of 10k
10000
9000
8000
7000
6000
5000
4000
0
0.25
0.5
0.75
1
Difference between upper & lower emission means
Geometric
2n-state Poisson
2-state Poisson

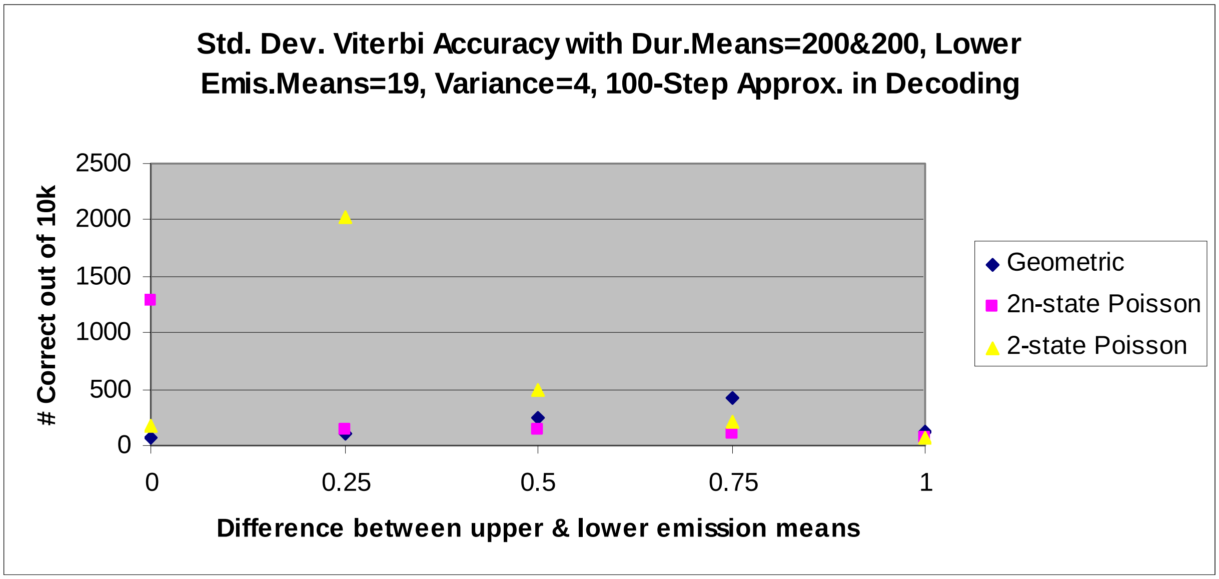
Std. Dev. Viterbi Accuracy with Dur.Means=200&200, Lower Emis.Means=19, Variance=4, 100-Step Approx. in Decoding
Correct out of 10k
2500
2000
1500
1000
500
0
0
0.25
0.5
0.75
1
Difference between upper & lower emission means
Geometric
2n-state Poisson
2-state Poisson
