# Supplementary material for: A novel, fast, HMM-with-Duration implementation – for application with a new, pattern recognition informed, nanopore detector
Source: BMC Bioinformatics. 2007 Nov 1;8(Suppl 7):S19. doi: 10.1186/1471-2105-8-S7-S19 (PMC2099487; doi:10.1186/1471-2105-8-S7-S19)
Supplement: Supplementary file 5 [file 1471-2105-8-S7-S19-S4.doc]

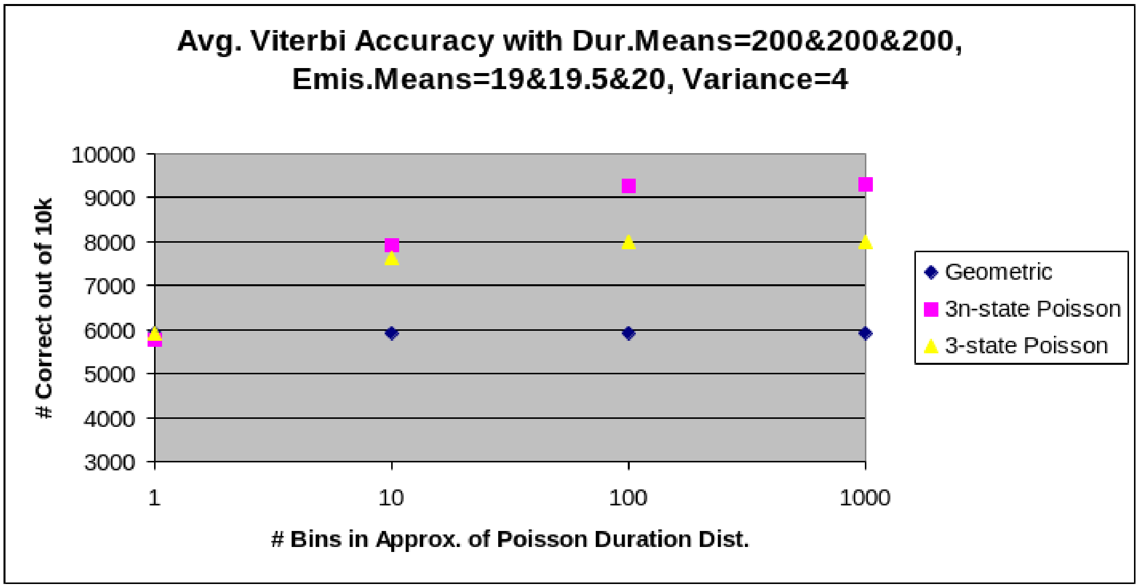
Avg. Viterbi Accuracy with Dur.Means=200&200&200,
Emis.Means=19&19.5&20, Variance=4
# Correct out of 10k
10000
9000
8000
7000
6000
5000
4000
3000
1
10
100
1000
# Bins in Approx. of Poisson Duration Dist.
Geometric
3n-state Poisson
3-state Poisson

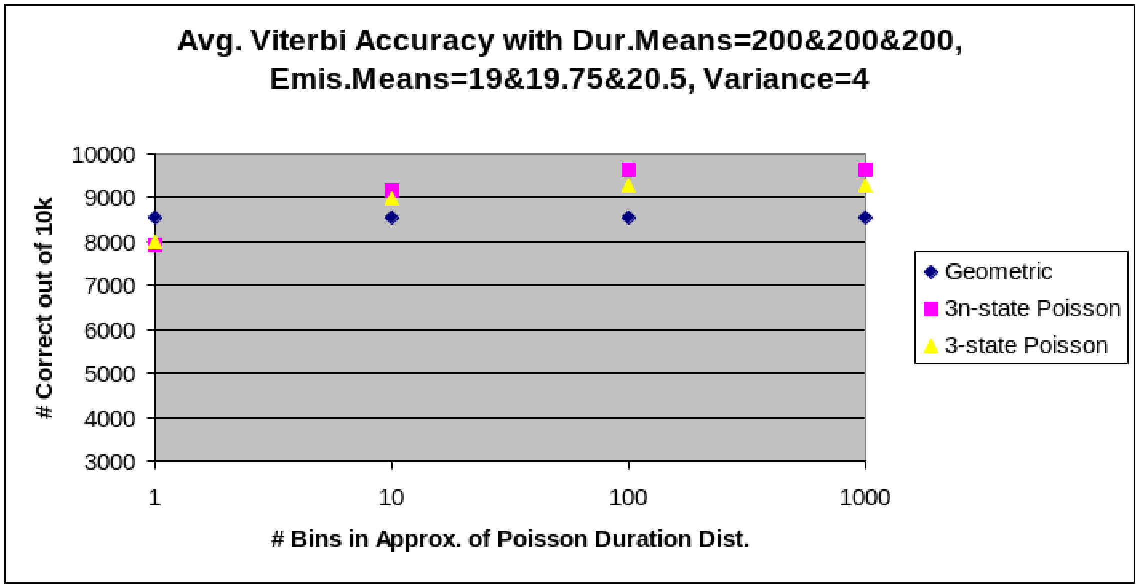
Avg. Viterbi Accuracy with Dur.Means=200&200&200,
Emis.Means=19&19.75&20.5, Variance=4
# Correct out of 10k
10000
9000
8000
7000
6000
5000
4000
3000
1
10
100
1000
# Bins in Approx. of Poisson Duration Dist.
Geometric
3n-state Poisson
3-state Poisson
