# Supplementary material for: A novel, fast, HMM-with-Duration implementation – for application with a new, pattern recognition informed, nanopore detector
Source: BMC Bioinformatics. 2007 Nov 1;8(Suppl 7):S19. doi: 10.1186/1471-2105-8-S7-S19 (PMC2099487; doi:10.1186/1471-2105-8-S7-S19)
Supplement: Supplementary file 6 [file 1471-2105-8-S7-S19-S8.doc]

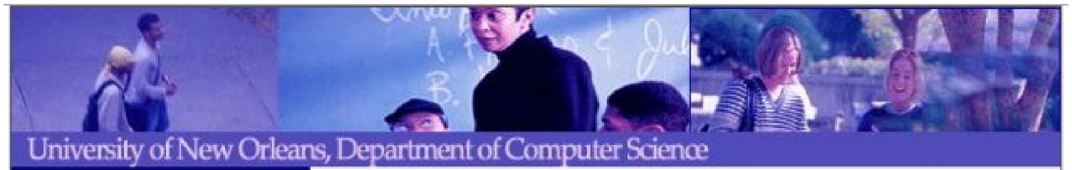

Home - The Biomedical Informatics Group at UNO

RIC Home - The Nanopore Biophysics/Cheminformatics Group at UNO

Channel Current Cheminformatics Architecture, Background, Literature

Discriminatory Resolution and Conformational properties of a set of DNA haripins

Antibody Conformational Change, Activation of Effector Function, and Antigen Binding

Transcription Factors, their binding sites, and analysis of the Transcriptome

Meet the group

CCC HMM

Annotation File:

[ ] Browse...

File Index Start: [ ]

File Index End: [ ]

Truncation Length: [ ]

The number of States:

50

Upper signal-range as Baseline:

70

Lower signal-range as Baseline:

20

Number of EM iterations:

5

EVA Factor:

1

E-mail:
Results will be sent to this address.

[ ]

Submit
